# Clinical profiles and referral patterns of infants attending Danish chiropractic clinics: a nationwide cross-sectional study

**DOI:** 10.1186/s12998-026-00629-z

**Published:** 2026-02-24

**Authors:** Freja Gomez Overgaard, Cathrine Hedegaard-Johansson, Lise Hestbaek

**Affiliations:** 1 Medical Spinal Research Unit, Spine Centre of Southern Denmark, University Hospital of Southern Denmark, Kolding, Denmark; 2 Department of Regional Health Research, University of Southern Denmark, Odense, Denmark; 3 Department of Chiropractic, Faculty of Medicine, Health and Human Science, Macquarie University, Sydney, Australia; 4 Kiropraktorerne WestLoft, Private Chiropractic Practice, Copenhagen, Denmark; 5 Center for Muscle and Joint Health, Department of Sports Science and Clinical Biomechanics, University of Southern Denmark, Odense, Denmark; 6 The Chiropractic Knowledge Hub, Odense, Denmark; 7 Hospital Lillebaelt, Sygehusvej 24, 6000 Kolding, Denmark

**Keywords:** Chiropractic, Infant, Musculoskeletal, Patient referral, Medical records, Healthcare utilization.

## Abstract

**Objective:**

To describe the primary complaints of infants presenting to Danish chiropractic clinics and explore the secondary complaints, and type of referrals from other healthcare professionals.

**Methods:**

A cross-sectional study based on routinely collected medical records from a national sample of chiropractic clinics in Denmark. Data were collected retrospectively in December 2019 and January 2020, covering infants (0–1 year) seen between November 2018 and October 2019. All 238 chiropractic clinics in the Danish Chiropractors’ Association were invited to participate via an electronic survey. The questionnaire captured demographic characteristics, primary and secondary complaints, symptom duration, and referral sources.

**Results:**

A total of 1,049 completed questionnaires were received. The most common primary complaint was abnormal range of motion (ROM) (48%), followed by crying/infantile colic (17%), feeding problems (5%), and disturbed sleep (5%). The majority of complaints had lasted between 1 week and 3 months, prior to consultation. Secondary complaints were reported in 58% of cases, most commonly abnormal ROM (41%), disturbed sleep (22%), and feeding problems (21%). Referrals were reported for 51% of infants, with healthcare visitors being the primary source (44%).

**Conclusion:**

This study provides insights into the clinical presentations and referral patterns of infants attending chiropractic clinics in Denmark. The findings highlight abnormal ROM as the predominant complaint, the central role of healthcare visitors in referrals, and the multifaceted nature of parental concerns. These results emphasize the need for evidence-based guidelines and stronger interdisciplinary collaboration to optimize infant care.

**Supplementary Information:**

The online version contains supplementary material available at 10.1186/s12998-026-00629-z.

## **Introduction**

Infants (children aged 0–1 year) constitute a large proportion of chiropractic patients under 18 years of age in Denmark [1] and the rest of Europe [2]. Over the past decade, there has been an increase in parents seeking chiropractic care for their infants in Denmark [3], reflecting changing healthcare utilization patterns. However, previous research into characteristics of clinical chiropractic practice for this age group is sparse and mainly dated before this increase in utilization. Therefore, the reasons behind these visits remain poorly understood.

Chiropractors in Denmark serve as primary care providers specializing in diagnosing and treating, and musculoskeletal disorders [4]. Although chiropractic services are partially subsidized by public health insurance, limited knowledge exists about infants’ clinical presentations and the factors that motivate parents to seek this care. As pointed out by the CAMbrella coordination action within the EU Framework Programme 7 [5], reviews show that we do not know enough about the circumstances in which Complementary and Alternative Medicine (CAM) is used by Europeans. In order to consider employing CAM as part of the solution to the health care, health creation and self-care challenges, it is vital to obtain a robust picture of CAM use [5]. This study will contribute a part of that puzzle by addressing a significant knowledge gap regarding infants in chiropractic care.

For this purpose, the present study aims to describe the clinical profiles of infants attending Danish chiropractic clinics. This information can contribute to optimizing healthcare delivery and resource allocation while supporting evidence-based practices. The study also aligns with calls for more research on chiropractors’ role in pediatric healthcare [6, 7]. Internationally, chiropractic care is also commonly sought for pediatric populations, including infants, for a range of musculoskeletal and non-musculoskeletal concerns. A scoping review of chiropractic utilization has shown that reasons for seeking chiropractic care among children are broadly consistent across regions, with musculoskeletal complaints being the most frequently reported, although patterns vary by age and healthcare context [8].

## Objectives

The main objective of this study is to identify the primary complaints of infants presenting to Danish chiropractic clinics. These will be described in terms of age, sex, and duration of complaint. Secondary objectives are to describe secondary complaints and type of referral from other healthcare professionals.

## Methods

### Study design and setting

This cross-sectional study is based on routinely collected medical records of 0-1-year-old patients from a national sample of chiropractic clinics. The data collection took place retrospectively during December 2019 and January 2020, including medical records from November 2018 to October 2019.

### Participants and recruitment

All 238 chiropractic clinics represented by the Danish Chiropractors’ Association were invited to participate. Invitations, including a link to an electronic questionnaire hosted on SurveyXact, were distributed via email by the Chiropractic Knowledge Hub in November 2019. A reminder email was sent after two weeks to enhance the response rate. Participation was voluntary and anonymous, and no identifiable information about clinics, chiropractors, or patients was collected. However, the clinic’s proportion of pediatric patients was recorded to describe the case mix regarding pediatric patient load.

### Questionnaire development and pilot testing

The questionnaire was adapted from a previously published national survey instrument developed to describe pediatric patients in Danish chiropractic practice, which demonstrated content validity and feasibility for use in clinical settings [1]. It was modified for clinicians to complete, based on patient records. The final instrument consisted of 14 questions and took approximately 10–15 min to complete. No alternative instruments were considered, as the aim was to ensure comparability with previous nationwide studies of pediatric chiropractic practice and to use an instrument specifically designed for clinician-reported data based on medical records.

Before data collection, the questionnaire was tested by three chiropractors experienced in pediatric care. The pilot testing evaluated the questions’ clarity, relevance, and feasibility. Feedback was minimal, with only minor linguistic adjustments made to improve clarity and wording of selected items. No changes were made to the content, structure, or response options of the questionnaire. The finalized questionnaire was distributed electronically via SurveyXact, allowing participants to use a paper version, returned in a prepaid envelope, if required.

### Data collection

To account for any seasonal variations in primary complaints, clinics were randomly assigned one month between November 2018 and October 2019 for data collection. During their assigned month, chiropractors completed one questionnaire per patient under one year of age. If a clinic was closed during the assigned month, data were collected for the following month.

### Variables

The questionnaire collected data on:

- Age of the child (categorized as: 0–12 weeks, 13–24 weeks, and 25–52 weeks).
- Sex of the child.
- Predefined primary and secondary complaints, including subcategories (e.g., Abnormal range of motion was subcategorized into favorite side, abnormal neck movement etc.). Abnormal range of motion was assessed clinically by the treating chiropractor based on routine examination, including visual observation and gentle assessment of active and passive movement; no instrument-based measurements were used.
- Duration of primary complaint prior to the first chiropractic consultation (categorized as: <1 week, 1–4 weeks, 1–3 months, > 3 months).
- Referral sources to the chiropractic clinic (e.g., healthcare visitors, general practitioners). Referral information was limited to sources referring the infant to the chiropractic clinic; referrals made by chiropractors to other healthcare providers were not captured.
- Estimated proportion of pediatric patients in the clinic (categorized as: 0–10%, 10–20%, 20–30%, > 30%).
- Number of chiropractic treatments received during the recorded course of care (as documented in the medical record at the time of questionnaire completion).

### Data analysis

Descriptive analyses were performed using STATA v.16.0 [9]. Frequencies and percentages were calculated for categorical variables, and mean values with standard deviations (SD) were reported for continuous variables. Primary complaints were presented by age, sex, and duration.

The reporting of this study follows the STROBE (Strengthening the Reporting of Observational Studies in Epidemiology) guidelines for cross-sectional studies, where applicable.

Analyses were conducted using available data only. Missing data were minimal and were not imputed; where cell counts were below five, results were suppressed to maintain anonymity.

### Ethical considerations

The Danish Agency for Patient Safety classified this study as a quality assurance project, exempting it from the requirement for written consent according to Sect.  42 d, subsection 2, number 2 of the Health Act. The project was approved by the legal department at the University of Southern Denmark (#10.779). Data were handled according to regulations by the Danish Data Protection Agency.

## Results

### Overview of respondents and clinics

A total of 1,049 completed questionnaires were received. Two clinics used paper versions of the questionnaire, and 11 clinics had no patients in the age group during their assigned month. Clinics with 0–10% of their patient load comprising infants accounted for the largest proportion of responses (34%), indicating a satisfactory spread of clinics (Table 1). Data were collected at the patient level, and as clinics could contribute more than one questionnaire, the number of participating clinics and a clinic-level response rate could not be determined.

**Table 1:** Number of infant patients by pediatric patient load in the clinics

Proportion of infant patients in the clinic	*N* (%)
0–10%	357 (34.1)
10–20%	287 (27.4)
20–30%	280 (26.7)
> 30%	123 (11.8)
Total	1,047 (100)

Responses were received for every month of the study period, supporting the representativeness of the findings. The highest response rate was in April 2019 (*n* = 196 (18.4%)) and the lowest in November 2018 (*n* = 18 (1.7%)).

### Characteristics of the children

The study population consisted of 57% boys, with a mean age of 12.4 weeks (SD: 9.3) and the youngest age group (0–12 weeks) represented 68% of the total sample (Table 2).

**Table 2:** Age and sex distribution of infant patients

Age group (weeks)	*N* (%)	Boys (%)	Girls (%)
0–12	710 (67.7)	59.2	40.8
13–24	192 (18.3)	56.8	43.2
25–52	147 (14.0)	50.3	49.7
Total	**1,049 (100)**	57.1	42.9

### Number of treatments

Of the 931 infants who had completed treatment at the time of recording, the number of treatments ranged from 0 to 17. The majority of infants (66%) received 2–5 treatments, with an average of 4.5 treatments (SD: 2.5). The number of treatments was described overall and was not analysed in relation to specific complaint types. A smaller proportion (15%) received only one treatment, while 1.7% received more than 10 treatments.

### Primary complaints

The most common primary complaint was abnormal range of motion (ROM), reported in 48% of cases, followed by crying/infantile colic (17%), feeding problems (5%), and disturbed sleep (5%). General examination with no specific complaint was the primary reason for consultation for 9% of the children (Table 3).

**Fig. 1:**
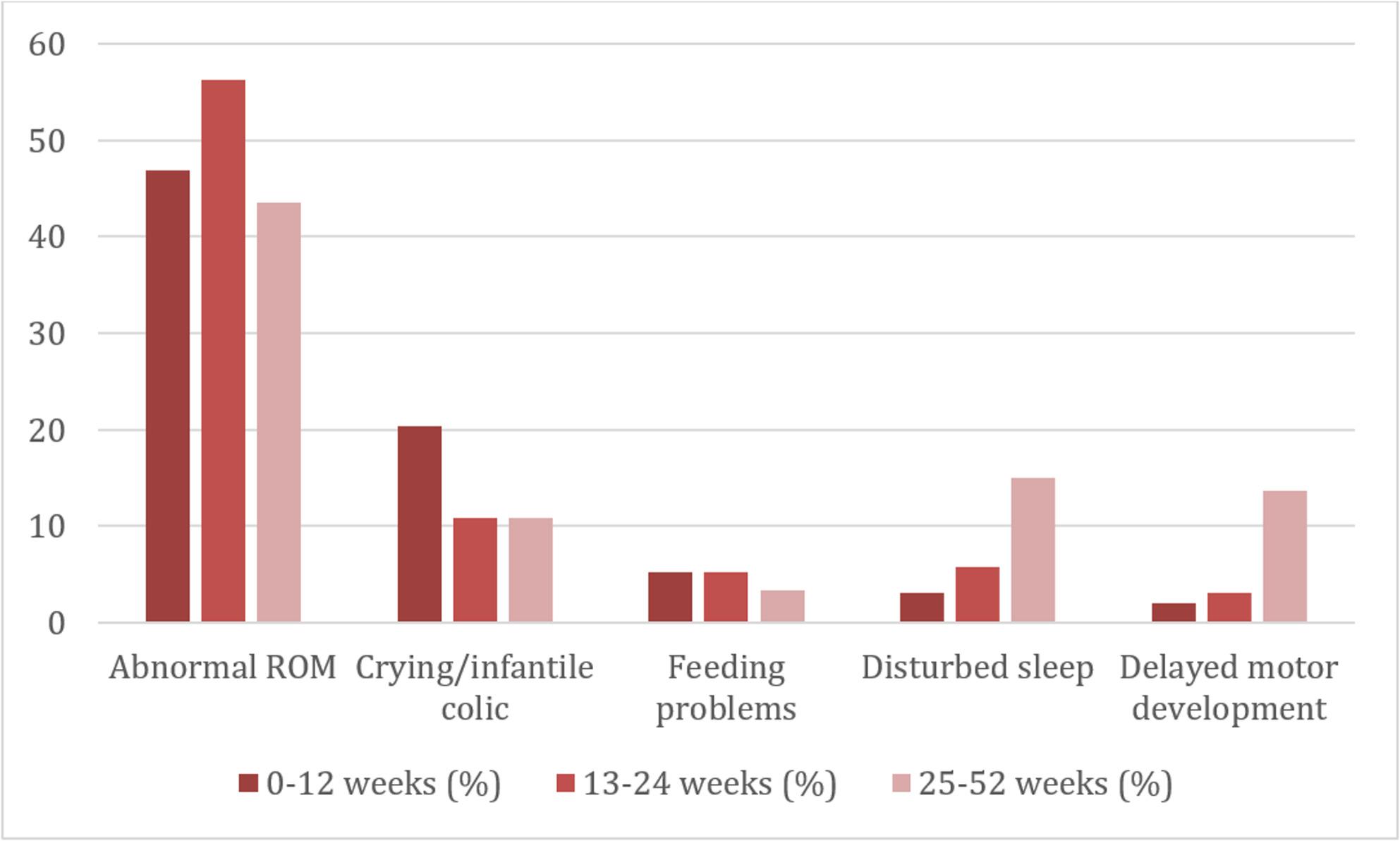
Five most common primary complaints by age group (%) among 1049 infants attending Danish chiropractic clinicsss

Age-specific patterns revealed that abnormal ROM was the leading complaint across all age groups, crying/colic was most common in the youngest infants (0–12 weeks), whereas disturbed sleep and delayed motor development were more frequently observed in older infants (25–52 weeks) (Fig. 1).

**Table 3:** Primary complaints of 1049 infants attending Danish chiropractic clinics. Described by age, sex and duration

Primary complaint	*n* (%)	Mean age in weeks (SD)	Sex, *n* (%)	Duration of primary complaint, %
Abnormal ROM	505 (48.1)	12.7 (10.0)	Boys: 58.5% Girls: 41.5%	< 1 week 3.8 1–4 weeks 37.2 1–3 months 42.2 > 3 months 16.4
Crying/infantile colic	181 (17.3)	9.7 (9.1)	Boys: 54.7% Girls: 45.3%	< 1 week 7.2 1–4 weeks 48.1 1–3 months 35.9 > 3 months 8.8
Feeding problems – latching	52 (5.0)	10.0 (9.4)	Boys: 51.9% Girls: 48.1%	< 1 week 11.5 1–4 weeks 42.3 1–3 months 40.4 > 3 months 5.8
Disturbed sleep	54 (5.1)	21.6 (14.8)	Boys: 51.9% Girls: 48.2%	< 1 week 3.7 1–4 weeks 38.9 1–3 months 27.8 > 3 months 27.8
Head deformity (asymmetry)	34 (3.2)	14.6 (11.0)	Boys: 58.8% Girls: 41.2%	< 1 week 0.0 1–4 weeks 26.5 1–3 months 44.1 > 3 months 29.4
Ear problems	0 (0.0)	-	-	-
Nose/throat problems	0 (0.0)	-	-	-
Gastrointestinal problems	34 (3.2)	8.5 (6.9)	Boys: 61.8% Girls: 38.2%	< 1 week 2.9 1–4 weeks 55.9 1–3 months 32.4 > 3 months 8.8
Regurgitation	6 (0.6)	9 (4.4)	Boys: 16.7% Girls: 83.3%	< 1 week 0.0 1–4 weeks 50.0 1–3 months 33.3 > 3 months 16.7
Reflux	5(0.5)	5.4 (2.3)	Boys: 40.0% Girls: 60.0%	-
Delayed motor development	40 (3.8)	24.2 (14.1)	Boys: 60% Girls: 40%	< 1 week 0.0 1–4 weeks 40.0 1–3 months 45.0 > 3 months 15.0
General examination	91 (8.7)	8.6 (8.7)	Boys: 58.4% Girls: 41.6%	< 1 week 2.2 1–4 weeks 0.0 1–3 months 2.2 > 3 months 0.0
Other	47 (4.5)	11.1 (11.5)	Boys: 59.6% Girls: 40.4%	< 1 week 2.1 1–4 weeks 59.6 1–3 months 29.8 > 3 months 8.5

Primary complaint: reflux *n* < 5,to maintain anonymity, the distribution of duration has not been specified

### Duration of primary complaints

For most conditions (abnormal ROM, crying/infantile colic, feeding problems, gastro-intestinal problems, and regurgitation) the vast majority had lasted from 1 week to 3 months prior to consultation. More children with disturbed sleep and head deformity reported long duration, with 28% and 29%, respectively, reporting symptoms for more than 3 months (Table 3). For most complaints (87%), the onset was not associated with trauma.

### Secondary complaints

Secondary complaints were reported in 58% of cases (*n* = 608). These were defined based on parental reports. The most common were abnormal ROM (41%), disturbed sleep (22%), and feeding problems (21%) (Table 4).

**Table 4:** Secondary complaints of 1049 infants attending Danish chiropractic clinics

Secondary complaint	*N* (%)
None	441 (42.0%)
Abnormal ROM	250 (23.8%)
Disturbed sleep	134 (12.8%)
Feeding problems	127 (12.1%)
Crying/infantile colic	108 (10.3%)
Gastrointestinal problems	111 (10.6%)
Head deformity	77 (7.3%)
Other	96 (9.2%)

### Referral sources

Referrals were reported for 51% of children, with healthcare visitors - publicly employed nurses providing preventive health services and routine follow-up to families with infants within the Danish healthcare system - being the most common source (44%), followed by family or friends (20%) (Table 5).

**Table 5:** Referral sources for infants attending Danish chiropractic clinics, who had some sort of referral (*n* = 535)

Referral source	*n* (%)
Healthcare visitor	235 (43.7%)
Family or friends	107 (19.9%)
Physiotherapist	63 (11.7%)
General practitioner	19 (3.5%)
Midwife	19 (3.5%)
Other	95 (17.7%)

### Subcategories of ROM complaints

Among infants with abnormal ROM, the most common subcategory was “favorite side,” reported in 75% of the primary ROM complaints. This was followed by “abnormal ROM in the cervical spine” (37%), “C-shape (skew position in the spine)” (27%), and “not fond of lying prone” (19%). These patterns were similar for secondary complaints (Appendix 1).

## Discussion

In this sample of infants attending chiropractic clinics in Denmark, 9% came for a general examination without a specific complaint. Among the rest, abnormal ROM was the dominant primary complaint followed by crying/infantile colic. These findings align with prior research identifying musculoskeletal issues as common reasons for chiropractic visits [1, 2].

Our findings are broadly consistent with international literature on pediatric chiropractic care. A scoping review by Béliveau et al. [8] identified musculoskeletal complaints as the most commonly reported reasons for seeking chiropractic care among children across multiple countries, alongside regulatory or functional concerns such as crying and sleep disturbances in younger age groups. While that review included heterogeneous pediatric populations and study designs, the predominance of musculoskeletal presentations aligns with the high proportion of abnormal range of motion observed in the present study.

However, differences in reported complaint profiles across studies may reflect variation in age distributions, healthcare systems, and data sources, including whether information is reported by parents or clinicians. The present study contributes infant-specific, clinician-reported data from a nationwide setting, thereby complementing existing international literature and providing more granular insight into early-life chiropractic consultations.

Notably, age-related patterns were observed, with crying/infantile colic being more prevalent among younger infants, whereas disturbed sleep and delayed motor development were more frequently reported in older infants. This likely reflects developmental trajectories during infancy, where parental concerns shift from early regulatory behaviours toward sleep and motor milestones as the child grows. These patterns underscore that chiropractic consultations in infancy are closely linked to age-specific parental concerns and developmental stages.

The higher prevalence of abnormal ROM in younger infants than in previous studies may reflect developmental factors or heightened parental concerns in early infancy [1, 2]. However, it could also be assigned to the fact that the complaints are chiropractor-reported, whereas previous studies were based on parental reporting [1]. Chiropractor-reported data may differ from parent-reported data. Clinicians are likely to identify movement asymmetries or restrictions during routine examination that may not be perceived by parents as discrete complaints, particularly in early infancy. Equally, parental reporting may place greater emphasis on behavioural or regulatory concerns, such as crying or sleep disturbances, which are more readily observable in everyday settings. These methodological differences may contribute to variation in the relative prevalence of specific complaint categories across studies and should be considered when comparing findings between clinician-reported and parent-reported data sources.

Secondary complaints, including disturbed sleep and feeding problems, was high in numbers suggesting that parents often seek chiropractic care for complex health concerns. These findings align with previous reports of chiropractors managing a wide range of complaints in pediatric populations [6].

Clinics varied substantially in their pediatric caseload, with the largest proportion of responses coming from clinics where infants comprised less than 10% of the total patient load. This suggests that the findings are not driven solely by clinics with a pediatric specialization, but rather reflect typical chiropractic practice across Denmark. It also indicates that even clinics with relatively limited pediatric caseloads commonly encounter infant patients with musculoskeletal concerns.

The large proportion of infants with healthcare visitors as referral source highlights the close relationship between new families and their healthcare visitors in the Danish health care system, as well as good communication between these and the chiropractors. However, the low referral rate from general practitioners suggests potential for improved interdisciplinary communication.

### Clinical implications

The findings highlight that infants presenting to chiropractic care often have musculoskeletal concerns complemented by additional regulatory or functional complaints, such as disturbed sleep or feeding problems. Chiropractors should therefore adopt a comprehensive assessment approach that considers both musculoskeletal findings and parental concerns, particularly in early infancy where abnormal range of motion is frequently identified.

Age-related differences in presenting complaints suggest that clinical focus may need to adapt across infancy, with increased attention to crying and regulatory issues in younger infants and motor development or sleep-related concerns in older infants. Awareness of these age-specific patterns may support more tailored communication with parents and more appropriate clinical prioritisation.

The important role of healthcare visitors as referral sources underscores the importance of clear communication and mutual understanding between chiropractors and other primary care providers involved in infant care. Strengthening interprofessional dialogue, regarding assessment findings, clinical reasoning, and follow-up, may support more coordinated care pathways and improve continuity of care for infants and their families.

### Strengths and limitations

This study uses a nationwide sample, randomization of data collection months to account for seasonal variations, and a structured, pilot-tested questionnaire. These factors increase the findings’ robustness and applicability to chiropractic care in Denmark but may not reflect pediatric chiropractic patients in other countries.

Participation by chiropractic clinics was voluntary, which may have introduced selection bias if participating clinics differed from non-participating clinics. The survey was conducted anonymously to comply with data protection regulations, which prevented evaluation of non-responders and assessment of clinic-level representativeness. Therefore, the extent to which the participating clinics reflect the broader population of Danish chiropractic clinics remains uncertain. However, chiropractors reporting 0–1-year-olds representing less than 10% of their patient load were responsible for the largest part of the responses (34%), indicating that pediatric specialty clinics do not dominate the dataset.

Data were collected retrospectively from routinely recorded medical records, and clinical complaints were reported by chiropractors without independent validation. This may have influenced the classification and prevalence of specific complaint categories. Referral data were limited to sources referring infants to chiropractic care, and referral patterns from chiropractors to other healthcare providers were not assessed. Additionally, the descriptive nature of the study precludes causal interpretation of observed patterns.

Finally, data collection occurred prior to the COVID-19 pandemic (2018–2019). While the study focuses on fundamental clinical presentations and referral sources in infancy, changes in healthcare utilization patterns following the pandemic cannot be excluded.

Future research should investigate the motivations and expectations of parents seeking chiropractic care for their infants as well as the long-term outcomes of such care. Additionally, studies evaluating the effectiveness of chiropractic interventions for common complaints such as abnormal range of motion and crying in infants are needed. Expanding interdisciplinary collaboration between chiropractors, healthcare visitors, general practitioners, and midwives could potentially enhance the quality of pediatric healthcare.

## Conclusion

This study provides valuable insights into the clinical presentations and referral patterns of infants attending chiropractic clinics in Denmark. The findings illustrate the prevalence of abnormal ROM as a primary complaint, the role of healthcare visitors in referrals, and the complex nature of parental concerns. These results underscore the need for evidence-based guidelines and enhanced interprofessional collaboration to improve care for this growing patient group.

## Supplementary Information

Below is the link to the electronic supplementary material.

Supplementary Material 1

Supplementary Material 2

## Data Availability

The data used and/or analyzed during the current study are available from Lise Hestbaek on reasonable request.

## Abbreviations

CAM: Complementary and Alternative Medicine
ROM: Range of motion
SD: Standard deviation
STROBE: Strengthening the Reporting of Observational Studies in Epidemiology)

## References

[1] Hestbaek L, Jørgensen A, Hartvigsen J. A Description of children and adolescents in danish chiropractic practice: results from a nationwide survey. J Manipulative Physiol Ther. 2009;32(8):607–15. 10.1016/j.jmpt.2009.08.024 19836596 10.1016/j.jmpt.2009.08.024

[2] Marchand AM Chiropractic care of children from birth to adolescence and classification of reported conditions: an internet Cross-Sectional survey of 956 European chiropractors. J Manipulative Physiol Ther. 2012;35(5):372–80. 10.1016/j.jmpt.2012.04.008. 22627100 10.1016/j.jmpt.2012.04.008

[3] Denmark S. Number of chiropractic visits for pediatric 0–12 months old in Denmark in the time period 2008–2018 [in Danish]. *Statistikbanken*, 2008–2018. Accessed 20 May 2020, Available from: https://www.statistikbanken.dk/statbank5a/default.asp?w=1882 (2020).

[4] *The Danish Health Act* (*Sundhedsloven*), Consolidation Act no. 210 of Jan. 27, 2022. Ministry of Health, Copenhagen, Denmark. Available from: https://www.retsinformation.dk/eli/lta/2022/210

[5] Fischer F, Lewith G, Witt CM, Linde K, von Ammon K, Cardini F, Falkenberg T, Fønnebø V, Johannessen H, Reiter B, Uehleke B, Weidenhammer W, Brinkhaus B. A research roadmap for complementary and alternative medicine – what we need to know by 2020. Forsch Komplementmed. 2014;21(2):e1–16. 10.1159/000360744 24851850 10.1159/000360744

[6] Alcantara J, Ohm J, Kunz D. The safety and effectiveness of pediatric chiropractic: a survey of chiropractors and parents in a practice-based research network. Explore, 5(5), 290–295, 2009. 10.1016/j.explore.2009.06.002 19733815 10.1016/j.explore.2009.06.002

[7] Kamper SJ, et al. What is the role of chiropractors in the management of musculoskeletal conditions? J Orthop Sports Phys Therapy. 2017;47(10):712–30. 10.2519/jospt.2017.7469. 10.2519/jospt.2017.7469 28918691

[8] Beliveau PJ, Wong JJ, Sutton DA, Simon NB, Bussières AE, Mior SA, French SD. The chiropractic profession: a scoping review of utilization rates, reasons for seeking care, patient profiles, and care provided. Chiropr Man Ther. 2017;25(1):35. 10.1186/s12998-017-0165-8 PMC5698931 29201346

[9] StataCorp. Stata statistical software: release 16. College Station, TX: StataCorp LLC; 2019.

