# Supplementary material for: Clinical profiles and referral patterns of infants attending Danish chiropractic clinics: a nationwide cross-sectional study
Source: Chiropr Man Therap. 2026 Feb 24;34:9. doi: 10.1186/s12998-026-00629-z (PMC13037122; doi:10.1186/s12998-026-00629-z)
Supplement: Supplementary file 1 — Supplementary Material 1 [file 12998_2026_629_MOESM1_ESM.docx]

Appendix:

| Secondary complaints among infants in Danish chiropractic clinics | |
| --- | --- |
| **Secondary complaint** | **N = 608 (100.0%)** |
| Abnormal ROM | 249 (41.0) |
| Crying/infantile colic | 108 (17.8) |
| Feeding problems – latching | 127 (20.9) |
| Disturbed sleep | 134 (22.0) |
| Head deformity (asymmetry) | 77 (12.7) |
| Ear problems | 7 (1.2) |
| Nose/throat problems | 8 (1.3) |
| Gastrointestinal problems | 111 (18.3) |
| Regurgitation | 61 (10.0) |
| Delayed Motor development | 81 (13.3) |
| General examination | 6 (1.0) |
| Reflux | 33 (5.4) |
| Other | 96 (15.8) |
| It was possible for the chiropractors to choose more than one subcategory for each child.  N = 608 since not all children were reported to have one or more secondary complaints. | |

| Subcategories of primary and secondary complaints among infants in Danish chiropractic clinics | | |
| --- | --- | --- |
| **Subcategories of complaint** | **Primary complaint**  **N = 1049 (100.0%)**  **n (%)** | **Secondary complaints**  **N = 608 (100.0%)**  **n (%)** |
| **Abnormal ROM** | 505 (48.1) | 250 (41.1) |
| Favourite side | 381 (75.4) | 143 (23.5) |
| Abnormal ROM in the cervical spine | 189 (37.4) | 111 (18.3) |
| Not fond of lying prone | 97 (19.2) | 64 (10.5) |
| C-shape (skew position in the spine) | 136 (26.9) | 60 (9.9) |
| SI-joints | 11 (2.2) | 21 (3.5) |
| Asymmetrical glutes/hips | 8 (1.6) | 18 (3.0) |
| Suspicion of hip dysplasia | 0 (0) | 4 (0.7) |
| Asymmetrical tonus in the back muscles | 11 (2.2) | 7 (1.2) |
| Other (describe) | 64 (12.7) | 0 (0) |
| **Head deformity (asymmetry)** | 34 (6.7) | 77 (12.7) |
| Plagiocephaly | 31 (6.1) | 66 (10.9) |
| Brachycephaly | 3 (0.6) | 5 (0.8) |
| **Motor development** | 40 (3.8) | 81 (13.3) |
| Not able to hold the head independently | 5 (1) | 20 (3.3) |
| Do not use arms and/or legs normally | 11 (2.2) | 32 (5.3) |
| Not able to turn from anterior to prone | 4 (0.8) | 11 (1.8) |
| Not able to turn from prone to anterior | 3 (0.6) | 6 (1.0) |
| Not able to crawl | 8 (1.6) | 4 (0.7) |
| Not able to be in a seated position independently | 2 (0.4) | 2 (0.3) |
| Not able to be in a standing position independently | 1 (0.2) | 1 (0.2) |
| Other (describe) | 21 (4.2) (not specified) | 27 (4.4) (not specified) |
