# Supplementary material for: Clinical profiles and referral patterns of infants attending Danish chiropractic clinics: a nationwide cross-sectional study
Source: Chiropr Man Therap. 2026 Feb 24;34:9. doi: 10.1186/s12998-026-00629-z (PMC13037122; doi:10.1186/s12998-026-00629-z)
Supplement: Supplementary file 2 — Supplementary Material 2 [file 12998_2026_629_MOESM2_ESM.pdf]

Chiropractic & Manual Therapies MANUSCRIPT REVISION CHECKLIST

Manuscript title: Clinical profiles and referral patterns of infants attending Danish chiropractic clinics: A nationwide cross-sectional study

First author name: Freja Gomez Overgaard

Upload this completed checklist with your manuscript in the editorial submission system

ITEM	Mark with X, provide page number, or write N/A
**Enter total word count** (main body text only, not including figures and tables)	N= 2792
**Enter Abstract word count (maximum 350 words)**	N= 236
**Abstract:** A full structured abstract is required for research articles and systematic reviews. Ensure the objectives and the results in the abstract correspond with those in the main text. Ensure the abstract in the main manuscript is the same as the abstract in the submission system.	X
**Article Processing Charges (APCs):** If your manuscript meets the criteria, you may apply for APC coverage by the journal partners. See here for details: https://chiromt.biomedcentral.com/criteria<br>Note that **you need to apply for this APC coverage *before* submitting your manuscript.**<br>For other possible APC coverage, see here for our publisher's information about discretionary waivers and discount policies for authors in financial need: https://www.springernature.com/gp/open-research/policies/journal-policies	X
**Study registration:** Have you pre-registered your study? If your study is a clinical trial, it *must* be pre-registered (before enrolment of the first participant) in a clinical trial registry and trial registration information should be included in the manuscript. Authors failing to prospectively register a clinical trial risk its inadmissibility to our journal. If your clinical trial was not registered prospectively, you must include the words 'retrospectively registered', and provide an explanation for why registration was delayed. With retrospective registration of a trial, your manuscript will only be considered under exceptional circumstances.<br>If your study is a systematic review, consider PROSPERO. For other study designs, consider the Open Science Framework, or other equivalent registries.	*N/A*
**Reporting guidelines:** Research manuscripts must conform with the appropriate reporting guidelines, where relevant. Checklists are available for a number of study designs, including: randomised controlled trials (CONSORT) and protocols (SPIRIT); systematic reviews (PRISMA); control interventions (COPPS statement); observational studies (STROBE); qualitative studies (COREQ); and case reports (CARE). This list is not exhaustive, so refer to the EQUATOR website for other reporting guidelines.	*STROBE*
If a **checklist** exists for the relevant reporting guideline for your manuscript, upload this with your submission.	**X**
**Ethics approval:** Include the name of the ethics committee(s) or institutional review board(s) and **the approval number(s)**. For further details of the journal's editorial policies and ethical guidelines see:<br>https://www.biomedcentral.com/getpublished/editorial-policies#ethics+and+consent	N/A
**Date of study/data collection:** Start and end dates must be included in **abstract and main manuscript text.**	X
**Patient consent:** For all manuscripts that include details, images, or videos relating to individual participants, written informed consent for the publication of these must be obtained from the participants (or their parent or legal guardian in the case of children under 16), and a statement to this effect should appear in the manuscript.	N/A
**References:**<br>• See: https://chiromt.biomedcentral.com/submission-guidelines/preparing-your-manuscript	X
• Ensure you cite the primary reference to support your statements. For example, cite a primary research article rather than a narrative review to support your assertions	X

Please complete items over the page

ITEM	Mark with X, provide page number, or write N/A
**Permission to reproduce items:** If any material has been previously published elsewhere, **permission must be obtained from the copyright owner** to reproduce the item in *Chiropractic & Manual Therapies*. Further, if you do not own the copyright of any image, text or other material in your manuscript, you MUST provide evidence of permission for publication under the creative commons licence, and attribute appropriately in the manuscript.	N/A
**Acknowledgements and "personal communications":** Authors require written permission from all those mentioned in the Acknowledgements section. Editors may request evidence of this.	X
**Competing interests:** Authors must disclose any financial and non-financial competing interests. Think of it this way: you should disclose any competing interests that may cause embarrassment if they become public after the publication of the manuscript. See: http://www.biomedcentral.com/getpublished/editorial-policies#competing+interests	X
**Copyediting service:** Non-native speakers of English may choose to use a copyediting service, or the editors may recommend this if there are language issues with the paper. See here under "Style and language": https://chiromt.biomedcentral.com/submission-guidelines/preparing-your-manuscript	X
**PROOF READ** the manuscript carefully and make any final changes. Chiropractic & Manual Therapies will NOT copyedit submitted manuscripts for style or language. Authors are advised to write clearly and simply and to have their article checked by colleagues before final submission.	X
**ALL authors must approve the submitted version of the manuscript**	X
